# Correlation of serum anti-Mullerian hormone with hormonal and environmental parameters in Brazilian climacteric women

**DOI:** 10.1038/s41598-022-15429-7

**Published:** 2022-07-14

**Authors:** Thiago Magalhães Gouvea, Laura Alves Cota e Souza, Angélica Alves Lima

**Affiliations:** 1 Programa de Pós-Graduação em Ciências Farmacêuticas (CiPharma), Escola de Farmácia, Universidade Federal de Ouro Preto, Ouro Preto, Minas Gerais CEP 35400-000 Brazil; 2 Departamento de Análises Clínicas (DEACL), Escola de Farmácia, Universidade Federal de Ouro Preto, Ouro Preto, Minas Gerais Brazil

**Keywords:** Biomarkers, Endocrinology

## Abstract

This study aimed to identify the correlation among anti-Mullerian Hormone serum levels and 25-OH-D, obesity, metabolic syndrome (MetS), and sexual hormones in climacteric women classified according to stages of reproductive aging (SRA). A cross-sectional study was conducted with a total of 177 Brazilian climacteric women between 40 and 64 years old. Concentrations of AMH were measured using the Access 2 Immunoassay System. A multiple linear regression analysis was used to identify the relationship among AMH, 25-OH-D, obesity, MetS, sexual hormones, sociodemographic and lifestyle factors. AMH levels decreased with increased age (B = − 0.059; p < 0.001), and reproductive aging (B = − 0.483; p < 0.001). Obesity indicators, lifestyle characters, 25-OH-D levels and MetS were not significantly associated with AMH serum concentration. Negative correlation was found for FSH (B = − 0.009; p < 0.001) and LH (B = − 0.006; p = 0.004); positive correlation for E2 (B = 0.001; p = 0.011), DHEAS (B = 0.003; p < 0.001) and SHBG (B = 0.003; p = 0.005). In the model adjusted for SRA, FSH levels (p < 0.001) and DHEAS (p = 0.014) were associated with AMH. Although, with the adjustment for age, only FSH remained with a significant association (p = 0.001). Of the other analytes, none was associated with AMH, regardless of the model fit. Our findings confirm that serum AMH level decreased with age and FSH levels, but there is no correlation between AMH with obesity, 25-OH-D, MetS or other sexual hormones in Brazilian climacteric women.

## Introduction

Anti-Mullerian hormone (AMH) is a homodimeric glycoprotein, member of the transforming growth factor beta (TGF-β) superfamily^1–3^. In adult women, AMH is produced and secreted exclusively by granulosa cells of antral and preantral follicles and play a fundamental role in ovarian folliculogenesis through paracrine and autocrine effects: repressing the recruitment, selection and maturation of follicles, by inhibiting the action of follicle-stimulating hormone (FSH)^4–8^.

Serum AMH levels increase until the third decade of life and slowly decline with increasing age. When the number of follicles drops dramatically by a few thousand, pattern of the menstrual cycle becomes irregular, with AMH becoming undetectable about 3–5 years before the follicle stock is exhausted, starting menopause^9–11^.

It exhibits a facility for clinical application in relation to other currently available markers of ovarian aging; such as FSH and estradiol (E2); since serum AMH concentration is independent of gonadotropins and remaining relatively constant throughout the menstrual cycle^3,12–14^.

AMH is also an important endocrine marker, used as a support criterion for the classification in stages of reproductive aging (SRA), according to Stages of Reproductive Aging Workshop (STRAW + 10). STRAW + 10 classifies adult woman's life in three phases of variable duration: reproductive, menopausal transition and postmenopausal. These phases include a total of seven steps centered around the final menstrual period (FMP, Stage 0, menopause). To define each stage, this classification is based mainly on menstrual criteria and some hormonal factors in addition to AMH, such as FSH and inhibin B, and the count of antral follicles (AFC)^15–17^.

In addition, AMH levels correlate with antral follicle count and there is evidence that it is currently the best parameter available to measure ovarian reserve under different clinical situations, and its use in the diagnosis of polycystic ovary syndrome (PCOS), primary ovarian failure and prediction of menopause is feasible^18–21^. However, an international standard is lacking, which limits the comparison between different AMH assays. In addition, there are endogenous and exogenous factors that influence serum AMH levels, which also affect the interpretation of AMH values in clinical medicine^22,23^.

Studies have reported that vitamin D (25-OH-D) can influence reproductive life of women, and that vitamin D deficiency was associated with low ovarian reserve^24–26^. Likewise, some studies have pointed out a significant relationship between low serum AMH levels and obesity^27–31^, as well as cardiovascular risk^30,32,33^, in some women's groups.

Despite several studies associating AMH to these variables, results still remain inconclusive in relation to vitamin D^34–36^, obesity^37–39^, metabolic syndrome (MetS)^40,41^ and cardiovascular risk^33,40,42,43^. In addition, studies evaluating serum AMH levels according to SRA by STRAW + 10 are still rare, mainly using the chemiluminescence methodology^44,45^.

Based on these reports, the purpose of this study was to identify hormonal and environmental parameters and other correlates of serum Anti-Mullerian Hormone (AMH) concentrations in Brazilian climacteric women classified according to Stages of Reproductive Aging (STRAW + 10).

## Materials and methods

### Study participants and data collection

This cross-sectional analysis included 177 women, aged between 40 and 64 years. A non-probabilistic technique of convenience sampling was used.

This study was performed in accordance with the Declaration of Helsinki and was approved by the Research Ethics Committee of Universidade Federal de Ouro Preto—CEP/UFOP, under protocol CAAE—0030.0.238.000-09. All subjects gave written, informed consent before inclusion in the study.

The volunteers were recruited, from Basic Health Units of the city of Ouro Preto, Minas Gerais, Brazil. The participants were recruited by active search, by invitation of the nurses, health agents or researchers.

Recruited participants were classified into 3 groups: late reproductive phase (LRP; n = 59), menopausal transition (MT; n = 58) and postmenopause (PM; n = 60). This classification was performed considering regularity of the menstrual cycles, date of the last menstrual period and serum concentration of FSH, according to Stages of Reproductive Aging Workshop—STRAW + 10^16,17^.

All participants were interviewed using questionnaires that included questions about sociodemographic, lifestyle and reproductive characteristics.

Body weight, height, body fat percentage (%BF), waist circumference (WC), and hip circumference were measured with use of standard methods, following recommendation of World Health Organization (WHO)^46^. BMI was calculated by dividing body weight (kg) by the square of height (m^2^). WC was measured as the smallest circumference between the rib margin and iliac crest, and hip circumference was measured as the maximum circumference between the waist and hip. Waist-to-hip ratio (WHR) and waist-to-height ratio (WHtR) was calculated by waist circumference divided by hip circumference and height, respectively^46^.

MetS was defined according to the harmonized definition proposed by Joint Interim Statement (JIS)^47^, in which a women is diagnosed with MetS if three or more of the following criteria are met: abdominal obesity (WC ≥ 80 cm), high triglycerides (TG) (≥ 150 mg/dL), low HDL-C (< 50 mg/dL), high fasting glucose (≥ 100 mg/dL), and increased blood pressure (BP ≥ 130/85 mmHg).

### AMH analysis

Blood samples were allowed to clot and were centrifuged using standard conditions (3000 rpm, 10 min) within 30 min after venipuncture.

Serum concentrations of AMH were measured using a commercially available immunoassay protocol validated, on the Access 2 Immunoassay System (Beckman-Coulter Inc., CA, USA). Access AMH assay is a simultaneous one-step sandwich chemiluminescence immunoassay. The capture antibody (F2B/12H) is already bound on paramagnetic particles and the second antibody (F2B/7A) is alkaline phosphatase labeled. The light production is directly proportional to the concentration of AMH in the sample. The amount of analyte in the sample is determined from a stored, six-point calibration curve^48^.

### Other hormonal, binding protein, and vitamin analysis

Serum follicle-stimulating hormone (FSH), luteinizing hormone (LH), estradiol (E2), testosterone (T), dehydroepiandrosterone sulfate (DHEAS), sex hormone-binding globulin (SHBG) and 25-hydroxyvitamin D (25-OH-D) levels were measured using a chemiluminescence-based immunometric assay on the Access 2 Immunoassay System (Beckman-Coulter Inc., CA, USA). Free Androgen Index (FAI) was calculated as the percentage ratio of total testosterone to SHBG levels, both in nmol/L.

### Statistical analysis

Data entry and statistical analyses were performed using Epi-Data software version 3.1 (www.epidata.dk/download.php) and SPSS version 20.0 (IBM, Armank, New York). Kolmogorov–Smirnov test was used to assess normality. The data are expressed as mean ± standard deviation; medians and interquartile range (IQR) or as n (%). Comparisons between SRA-groups were performed using 1-way analysis of variance (ANOVA), *Kruskal–Wallis* or *Mann–Whitney* Test. A multiple linear regression analysis was used to identify the relationship among AMH, 25-OH-D, obesity, metabolic syndrome, sexual hormones, and other sociodemographic and lifestyle factors. In all tests, p < 0.05 was considered statistically significant.

### Ethics approval and consent to participate

This study was approved by the Research Ethics Committee of Universidade Federal de Ouro Preto—CEP/UFOP, under protocol CAAE—0030.0.238.000-09. All subjects gave written, informed consent before inclusion in the study.

## Results

Table 1 presents baseline characteristics of participants according to SRA.

**Table 1:** Baseline characteristics of research subjects, according to SRA (n = 177).

	SRA (STRAW + 10): *mean (SD), median (IQR), or n (%)*				
	LRP (n = 59)	MT (n = 58)	PM (n = 60)	Total	p value
**Age (years)**					
40 a 44	31 (52.5)	11 (19.0)	0	42 (23.7)	< 0.001
45 a 49	22 (37.3)	20 (34.5)	3 (5.0)	45 (25.4)	
50 a 54	6 (10.2)	20 (34.5)	15 (25.0)	41 (23.2)	
55 a 59	0	7 (12.1)	34 (56.7)	41 (23.2)	
60 a 65	0	0	7 (13.3)	8 (4.5)	
**Education level (years at school)**					
0 to 8 y	9 (15.3)	14 (24.1)	6 (10.2)	29 (16.4)	0.112
8 or more	50 (84.7)	44 (75.9)	54 (89.8)	148 (83.6)	
**Marital status**					
No partner	21 (35.6)	25 (43.1)	22 (36.7)	68 (38.4)	0.665
With partner	38 (64.4)	33 (56.9)	38 (63.3)	109 (61.6)	
Family income (MW)*					
0 to 1,00	17 (29.9)	26 (42.6)	9 (15.3)	52 (29.4)	0.103
1,01 to 2,00	24 (42.1)	17 (27.9)	22 (37.3)	63 (35.6)	
2,00 or more	15 (26.3)	17 (27.8)	27 (45.8)	59 (33.3)	
N.R	1 (1.8)	1 (1.6)	1 (1.7)	3 (1.7)	
**Tabagism**					
Yes	4 (6.8)	2 (3.4)	8 (13.3)	14 (7.9)	0.128
No	55 (93.2)	56 (96.6)	52 (86.7)	163 (92.1)	
**Alcohol intake**					
Yes	1 (1.7)	3 (5.2)	0	4 (2.3)	0.157
No	58 (98.3)	55 (94.8)	60 (100.0)	173 (97.7)	
**Regular physical activity**					
Yes	36 (61.0)	31 (53.4)	41 (68.3)	108 (61.0)	0.253
No	23 (39.0)	27 (46.6)	19 (31.7)	69 (39.0)	
**Sexual activity**					
Yes	47 (83.1)	50 (81.0)	44 (75.0)	141 (79.7)	0.525
No	10 (16.9)	11 (19.0)	15 (25.0)	36 (20.3)	
**Metabolic syndrome**					
Yes	16 (27.1)	17 (29.3)	16 (26.7)	49 (27.7)	0.943
No	43 (72.9)	41 (70.7)	44 (73.3)	128 (72.3)	
25-OH-D	24.0 (± 7.3) ^b^	26.4 (± 9.4)	28.6 (± 8.7)	26.4 (± 8.7)	0.014
WC (cm)	89.1 (± 12.2)	87.6 (± 12.8)	85.2 (± 9.3)	87.3 (± 11.6)	0.186
WHR	0.86 (± 0.09)	0.86 (± 0.08)	0.87 (± 0.06)	0.86 (± 0.08)	0.358
BMI (kg/m^2^)	26.7 (± 4.9) ^b^	26.1 (± 5.0) ^b^	24.1 (± 3.6)	25.6 (± 4.6)	0.004
BF (%)	35.2 (± 8.3) ^b^	33.4 (± 8.4)	30.7 (± 6.8)	33.1 (± 8.0)	0.009
WHtR	0.55 (± 0.08)	0.55 (± 0.09)	0.53 (± 0.06)	0.55 (± 0.08)	0.350
FSH (µUI/mL)	6.10^a.b^ (3.26–7.99)	23.09 ^b^ (7.54–69.60)	56.40 (41.10–83.81)	21.45 (6.45–59.53)	< 0.001
LH (µUI/mL)	7.45^a.b^ (4.40–14.19)	43.29 ^b^ (12.67–55.08)	45.81 (33.21–70.70)	32.3 (8.58–53.71)	< 0.001
E2 (pg/mL)	142^a.b^ (65–226)	63^b^ (21–174)	40 (25–56)	58 (31–162)	< 0.001
T (ng/dL)	33.85 (22.16–47.69)	27.76 (13.63–39.08)	27.40 (13.14–40.09)	29.8 (15.76–41.30)	0.096
DHEAS (µg/dL)	94.5^b^ (61.9–141.4)	81.6^b^ (53.8–108.9)	60.6 (30.3–100.7)	76.3 (49.10–113.60)	< 0.001
SHBG (nmol/L)	76.6^a.b^ (50.8–87.3)	51.9 (33.7–75.5)	56.6 (33.5–74.0)	60.5 (38.1–79.4)	0.001
FAI (%)	1.55 (0.77–2.43)	1.83 (0.77–2.80)	1.71 (0.73–3.01)	1.70 (0.75–2.72)	0.169
AMH (ng/mL)	0.454^a.b^ (0.132–1.428)	0.025^b^ (0.011–0.129)	0.009 (0.007–0.014)	0.028 (0.010–0.264)	< 0.001

*The minimum wages (MW) during data collection varied from R$ 724.00 to 880.00 (2014/2016); ^a,b^One-way ANOVA or Mann–Whitney Test; ^a^compared to the menopausal transition (MT); ^b^compared to the postmenopause (PM). Reference values for postmenopausal women: (FSH > 25.0 μUI/mL; LH 10.87–58.64 μUI/mL; E2 < 40 pg/mL; T < 75.0 ng/dL; DHEAS 8.0–231.0 μg/dL; SHBG 16.8–125.2 nmol/L and AMH 0.01–2.99 ng/mL).

The mean age of the study population was 50.1 ± 6.2 years, with a range of 40–64 years. Most women studied for 8 years or more (83.6%), lived with a partner (61.6%) and had low family income (65.0%). Alcohol consumption was reported by 2.3% and tabagism by 7.9%. Sedentariness was present in 39.0% of the sample. Most participants had sexual activity (79.7%). The prevalence of MetS was 2.1%. Mean WC, WHR and WHtR were 87.3 cm, 0.86 and 0.55, respectively. No association was found between SRA and years at school (p = 0.112), marital status (p = 0.665), family income (p = 0.103), tabagism (p = 0.128), alcohol intake (p = 0.157), regular physical activity (p = 0.253) or sexual activity (p = 0.525).

There was significant difference between SRA and 25-OH-D (p = 0.014), BMI (p = 0.004) and BF (p = 0.009). Serum 25-OH-D levels were statistically lower in LRP than the PM. For anthropometric parameters, %BF values were significantly higher in LRP than the PM, as well as BMI measurements that were also higher in both LRP and TM, compared to PM.

FSH and LH showed statistically significant difference between PM and the previous phase (p < 0.001, for all). E2 decreases as from MT, reaching its lowest level in PM, yielding statistically significant differences between this phase and the earlier stages (p < 0.001, for both). SHBG levels were statistically higher in LRP than the MT and PM (p = 0.001). The levels of DHEAS were also higher on LRP and MT, with a statistically significant difference when compared to PM (p < 0.001). No association was found between SRA and T (p = 0.096) or FAI (p = 0.169).

AMH mean level was 0.433 ± 0.935 ng/mL, with range 0.003–5.998, median 0.028. Its levels were statistically higher in late reproductive phase (LRP) than the menopausal transition (MT) and postmenopause (PM) (p < 0.001) (Table 1).

### Correlation between AMH and lifestyle/environmental factors

As expected, we observed significantly lower AMH concentrations among older women. The serum AMH level decreased with increased age (B = − 0.059; p < 0.001), and reproductive aging (B = − 0.483; p < 0.001). Obesity (WC, BMI, %BF), tabagism, alcohol intake, physical regular exercise, 25-OH-D levels and MetS were not significantly associated with AMH serum concentration (Table 2).

**Table 2:** Regression coefficients between lifestyle/environmental factors and serum AMH.

Log10 AMH	B	T	p value
Age	− 0.059	− 5.484	< 0.001
SRA	− 0.483	− 5.781	< 0.001
WC	0.009	1.273	0.205
BMI	− 0.010	− 0.487	0.627
BF%	− 0.007	− 0.708	0.480
Tabagism	0.058	0.368	0.714
Alcohol intake	− 0.496	− 1.753	0.081
Regular exercise	0.021	0.236	0.814
25-OH-D	− 0.004	− 0.733	0.465
MeTS	− 0.072	− 0.678	0.499
Intercept	2.960	5.714	< 0.001

Overall R^2^ = 0.656 (corrected R^2^ = 0.635).

The correlations can be seen in Table 3. There was none parameter that showed significant correlation with log_10_AMH, regardless of the model.

**Table 3:** Linear regression analysis of lifestyle/environmental factors with log_10_AMH in participants (n = 177).

	Model 1^a^		Model 2^b^	
	B ± SE	p value	B ± SE	p value
WC	0.006 ± 0.008	0.469	0.009 ± 0.007	0.205
BMI	− 0.011 ± 0.022	0.632	− 0.010 ± 0.021	0.627
BF%	− 0.004 ± 0.011	0.683	− 0.007 ± 0.010	0.480
Tabagism	− 0.031 ± 0.170	0.858	0.058 ± 0.158	0.714
Alcohol intake	− 0.573 ± 0.306	0.063	− 0.496 ± 0.283	0.081
Regular exercise	− 0.066 ± 0.095	0.486	0.021 ± 0.083	0.814
25-OH-D	− 5.991E-5 ± 0.005	0.991	− 0.004 ± 0.005	0.465
MeTS	− 0.145 ± 0.114	0.205	− 0.072 ± 0.106	0.499

^a^Model 1—adjusted for SRA.

^b^Model 2—adjusted for SRA and age.

### Correlation between AMH and sexual hormones

About sexual hormonal variables and AMH levels, a negative correlation was found for FSH (B = − 0.009; p < 0.001) and LH (B = − 0.006; p = 0.004). Showed positive relationship E2 (B = 0.001; p = 0.011), DHEAS (B = 0.003; p < 0.001) and SHBG (B = 0.003; p = 0.005). Only T (B = 0.001; p = 0.094) no presented correlation with the serum AMH levels (Table 4).

**Table 4:** Regression coefficients between sexual hormones and serum AMH.

Log10 AMH	B	T	p value
FSH	− 0.009	− 4.831	< 0.001
LH	− 0.006	− 2.924	0.004
E2	0.001	2.567	0.011
T	0.001	1.684	0.094
DHEAS	0.003	3.564	< 0.001
SHBG	0.003	2.819	0.005
Intercept	− 1.321	− 7.882	< 0.001

Overall R2 = 0.568 (corrected R2 = 0.553).

Table 5 shows the results of multiple regression analysis between sex hormones and log_10_ AMH levels. In the model adjusted for SRA, FSH levels (p < 0.001) and DHEAS (p = 0.014) were associated with AMH. However, with the adjustment for age, only FSH remained with a significant association (p = 0.001). Of the other analytes, none was associated with AMH, regardless of the model fit.

**Table 5:** Linear regression analysis of sexual hormones in relation with log_10_AMH in participants (n = 177).

	Model 1^a^		Model 2^b^	
	B ± SE	p value	B ± SE	p value
FSH	− 0.006 ± 0.002	< 0.001	− 0.005 ± 0.001	0.001
LH	− 0.003 ± 0.002	0.071	− 0.003 ± 0.002	0.074
E2	0	0.403	0	0.647
T	0	0.214	0	0.061
DHEAS	0.002 ± 0.001	0.014	0.001 ± 0.001	0.137
SHBG	0.002 ± 0.001	0.071	0.001 ± 0.001	0.164

^a^Model 1—adjusted for SRA.

^b^Model 2—adjusted for SRA and age.

## Discussion

Since the early 2000s, studies indicate that AMH levels have progressively decreased with increasing age in women, proving to be a good endocrine marker of ovarian reserve^19–21^. Ovarian aging is related to the decline in the quantity and quality of ovarian follicle pool^3,49^.

In this study, ANOVA revealed that there were significant differences in the levels of AMH among the SRA-groups: LRP, MT and PM (p < 0.001), which had already been reported by other studies^50–53^.

A long-term follow-up study conducted with 257 normovulatory women (21–46 years) found that AMH can significantly help predict menopause in an age-adjusted model^54^. Therefore, the detection of low serum AMH concentrations may be helpful for the prediction of ovarian reserve, onset of menopause, future fertility, and management of reproductive health^53,55,56^.

Additionally, we found that the mean AMH level (0.433 ± 0.935 ng/mL), in samples of Brazilian climacteric women, was lower than that reported in Chinese healthy women aged between 40 and 55 years (1.52 ± 1.88 ng/mL) by Du and cols^28^ and Tehrani and cols^57^ in Iranian women with mean age close to 40 years (1.65 ± 1.81 ng/mL), who measured AMH levels using the Beckman Coulter ELISA Gen II assay. However, this may mainly be because the methodology used in our study was chemiluminescence, unlike previous ones.

### Correlation between AMH and lifestyle/environmental factors

Our findings align with the other studies that reported an inverse association between age and AMH concentrations. It is well established that serum AMH concentration decreases progressively with increasing age. The follicle pool determined at birth decreases with age, which results in a decline in the total number of follicles that produce AMH^3,28,58–60^.

In this study, we were unable to observe a significant association between obesity and AMH concentration. This was also a finding in other studies^37,61^, that included only premenopausal and/or late reproductive age.

Many authors point out a significant association between low levels of AMH and obesity^27–31,62^, but this disagreement may be because the women studied are not in menopause, belong to specific groups (infertile, pregnant or PCOS patients) or the methodology used to determine AMH was not the same as in this study.

Vitamin D is a steroid hormone that acts through the transcription factor of nuclear genes, and the interest in understanding the roles it can play in female reproductive health is notorious^63^. In our study, we were not able to verify a statistically significant correlation between serum levels of AMH and 25-OH-D. Furthermore, only 7.9% (n = 14) were using exogenous vitamin D supplementation.

There are also articles in the literature that did not demonstrate any relationship between 25-OH-D and AMH, in climacteric women^41,64^. Otherwise, some studies found a positive relationship between 25-OH-D status and AMH at the cellular level^25^ and at the serum level^65^. In general, most null studies have sampled women from fertility clinic populations or those with PCOS, which may partly explain the negative results. Other factors that affect sun exposure and should be considered are geographic location and seasonality of collection.

At the cellular level, was reported that promoter region for the AMH gene contains a domain for vitamin D response element (VDRE). Vitamin D, via these response elements, modulates AMH expression directly^25,66^. Given this scenario, 25-OH-D deficiency should be considered when using serum AMH levels for the clinical diagnosis of menopausal prediction^67^. Therefore, such conflicting results explain the need for more research to determine the effect of vitamin D on AMH levels^68^.

Regarding AMH and cardiovascular risk, we did not find any correlation between serum AMH and MetS levels, adding another negative result in this research subject. Studies have not found a definite correlation between AMH and metabolic risk factors^41,69^.

However, some studies^33,43,70^ have shown that most changes in anthropometric, laboratory and other risk factors for CVD and MS seem to occur in transition period from the reproductive to the non-reproductive stage known as menopausal transition and do not include postmenopausal women, as well as this study. Therefore, menopause and age are factors that may predispose women to the development of MetS^41,71,72^. Thus, further studies are needed to unravel the temporal and causal relationship between the decrease in ovarian reserve and the increase in cardiovascular risk in women^40^.

### Correlations between AMH and Sexual hormones

Our results point to a negative correlation with log_10_ AMH concentration for FSH and LH; and positive for E2, DHEAS and SHBG. Only T did not show any type of correlation. In a model adjusted by SRA, levels of FSH and DHEAS were associated with AMH concentration. However, with the addition of variable age to the model, only FSH maintained correlation significantly.

Reports on correlations between AMH and other hormones may be inconsistent^73^.

A population-based study of Chinese women showed that AMH level was positively correlated with T and LH; negatively to FSH and was not significantly associated with E2^28^, same findings of La Marca and cols^20^, in women in LRP. Subsequently, other work with young women with menstrual cycle found significant inverse correlations between AMH and FSH in the early follicular phase, but there was no significant correlation with LH^74^, unlike our study. Other study in late-reproductive-aged women also showed negative correlation with AMH and FSH^65^. Finally, another Chinese paper studying women with and without polycystic ovary syndrome found that AMH level was positively correlated the LH, negatively associated with FSH, while they found that AMH was not significantly associated with T^75^, results partially in agreement with ours, despite LH.

Such findings are physiologically justified because with ovarian aging, the number of follicles decreases, as does their functional capacity, requiring high levels of FSH to reach follicular maturity and ovulation. As anovulation usually occurs, these changes occur as disturbances in the menstrual cycle. A paradoxical phenomenon at this stage is that an excessively high peak in LH secretion occasionally occurs^76,77^. During perimenopause, ovarian function appears to be highly variable. Hormone levels can vary during this period, with serum E2 concentration decreasing progressively, since it is produced by ovarian follicles, which can lead to appearance of symptoms in short and long term^78^.

In this study, relationship between AMH and FSH was confirmed, where we show a strong negative correlation, even after the models are adjusted for SRA and age. The aging ovary produces smaller amounts of AMH which leads to a rise in FSH on early follicular phase^79^. La Marca and cols^20^ postulated that it is not possible to establish whether the correlation between FSH and AMH reflects a direct physiological link between the two hormones, but FSH may have a negative role in the production of AMH by the ovary. Part of this association may have been influenced by sample of the population that was using oral contraceptives, since they may have an influence on FSH. In particular oral contraceptives suppress FSH and reduce size of the ovary, which can decrease follicle recruitment and functioning, result in a smaller number and size of antral follicle, which could modify serum AMH concentrations^61,80^.

Our non-significant results regarding the correlation with testosterone are consistent with other studies^61,81–83^.

Very few studies are found with previously examined associations between AMH and DHEAS. One does not find a significant correlation studying Korean climacteric women, mostly in late premenopausal phase^61^. In another, a meta-analysis shows that general evidence about the possible role of DHEA supplements as an adjuvant to improve the ovarian response was inconclusive^84^.

The evidence for association between SHBG and AMH has been inconsistent. Although we observed a positive association as well as Jung and cols^61^, others did not report an association^83^ or verified a significant inverse association^85^.

This study has some limitations, such as cross-sectional model that undermines the notion of causality in associations. Another limitation is reduced sample size.

However, there are several strengths in the study. We examined the association of AMH concentrations with several factors, including sociodemographic, lifestyle, environmental and hormonal factors, adjusting the results for important confounding factors such as age and stages of reproductive aging. In addition, the analysis of AMH concentrations was made by chemiluminescence, one of the most modern methodology available today. All samples were analyzed in a single laboratory, using an assay with excellent sensitivity with a limit of detection (LoD) of ≤ 0.02 ng/mL and a limit of quantitation (LoQ) of ≤ 0.08 ng/mL, demonstrated good validity and reproducibility^44,45^. To our knowledge, this is the first study on the relationship between serum AMH concentration with environmental, lifestyle and laboratory variables (25-OH-D, obesity, MetS, sexual hormones), in Brazilian climacteric women classified according to SRA.

In conclusion, our result pointed out there is no correlation between AMH with obesity, 25-OH-D, MetS or sexual hormones, except FSH, evaluated in Brazilian climacteric women.

## Data Availability

The datasets used and/or analysed during the current study available from the corresponding author on reasonable request.
